# The Effects of Magnetic Field Alignment on Lithium Ion Transport in a Polymer Electrolyte Membrane with Lamellar Morphology

**DOI:** 10.3390/polym11050887

**Published:** 2019-05-15

**Authors:** Pawel W. Majewski, Manesh Gopinadhan, Chinedum O. Osuji

**Affiliations:** 1 Department of Chemistry, University of Warsaw, 02098 Warsaw, Poland; pmajewski@chem.uw.edu.pl; 2 Department of Chemical and Environmental Engineering, Yale University, New Haven, CT 06511, USA; maneshphysik@gmail.com

**Keywords:** block copolymers, polyelectrolyte membrane, lithium transport, directed self-assembly, charge transport, magnetic alignment

## Abstract

The transport properties of block copolymer-derived polymer electrolyte membranes (PEMs) are sensitive to microstructural disorder originating in the randomly oriented microdomains produced during uncontrolled self-assembly by microphase separation. This microstructural disorder can negatively impact performance due to the presence of conductivity-impeding grain boundaries and the resulting tortuosity of transport pathways. We use magnetic fields to control the orientational order of Li-doped lamellar polyethylene oxide (PEO) microdomains in a liquid crystalline diblock copolymer over large length scales (>3 mm). Microdomain alignment results in an increase in the conductivity of the membrane, but the improvement relative to non-aligned samples is modest, and limited to roughly 50% in the best cases. This limited increase is in stark contrast to the order of magnitude improvement observed for magnetically aligned cylindrical microdomains of PEO. Further, the temperature dependence of the conductivity of lamellar microdomains is seemingly insensitive to the order-disorder phase transition, again in marked contrast to the behavior of cylinder-forming materials. The data are confronted with theoretical predictions of the microstructural model developed by Sax and Ottino. The disparity between the conductivity enhancements obtained by domain alignment of cylindrical and lamellar systems is rationalized in terms of the comparative ease of percolation due to the intersection of randomly oriented lamellar domains (2D sheets) versus the quasi-1D cylindrical domains. These results have important implications for the development of methods to maximize PEM conductivity in electrochemical devices, including batteries.

## 1. Introduction

Self-assembly of block copolymers (BCPs) is a fascinating phenomenon that can be utilized to create composite materials with periodic nanostructure in a cost-effective manner [1]. Easy access to nanometer-scale features coupled with compositional variety and thus tunable physical properties makes these nanoscale heterogeneous materials excellent candidates for selective transport applications including ion-conduction [2,3,4,5], pervaporation [6], and nanofiltration [7,8,9,10,11]. Ion conducting diblock copolymers are particularly compelling materials in the design of electrolyte membranes for rechargeable lithium batteries [12,13]. In this application, a polar polymer block acts as solid electrolyte facilitating lithium transport during the operation of the battery while the other block contributes to the mechanical integrity required for the membrane as a whole. Compared to homopolymer-based or porous electrolyte-soaked composite materials, block copolymer membranes offer better structural stability, hence potentially longer battery lifetime and greater safety of use. In particular, BCP membranes are promising for their potential to mechanically restrict the highly undesirable dendritic lithium formation during the charging process [14]. It is notable however that suppression of such dendrite formation has also been observed recently in relatively low modulus materials as well [15].

Engineering high performance block copolymer-based ion conducting membranes requires a theoretical framework describing charge transport in these materials. Interest in predicting the effective conductivity of heterogeneous media dates at least as far back as the theoretical studies by Maxwell [16] and Rayleigh [17]. The effective (ionic) conductivity in heterogeneous media can be described by various models including the percolation and the effective medium theories developed for diffusion of small molecules in polymer blends. The applicability of such models to a particular block copolymer system depends on the molar mass of the constituent blocks, the degree of block segregation (i.e., the magnitude of the block interaction parameter), the presence of ionic species, and system temperature. The microstructural model of Sax and Ottino [18] has proved useful in considering ion transport in strongly-segregated systems with well-developed morphology, such as amphiphilic block copolymers [2]. Such models are extensions of the effective medium theory approach and entail a combination of conductivity relations (for transport along pathways arranged in parallel and in series) with a statistical distribution of microdomain orientations. In its general form, the model also accounts for uneven sequestration and mobility of transported species in both microdomains. Taking a limiting assumption that conducting ions are solubilized only in one of the blocks and/or that their mobility in the second block is zero, we effectively limit all charge transport processes to one microdomain surrounded by an ideal insulator.

### 1.1. Microstructural Conductivity Model

**Figure 1:**
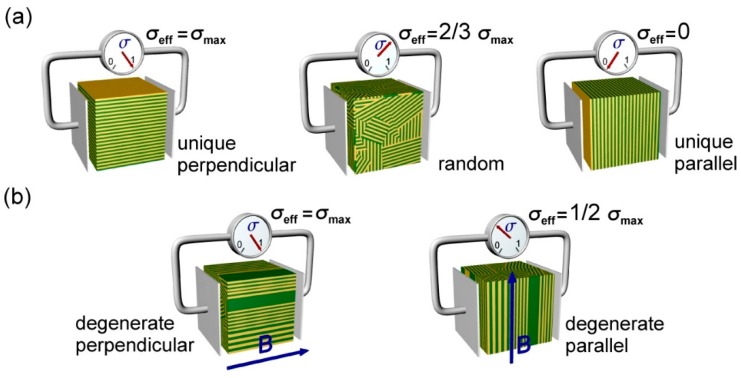
Ionic conductivity in a lamellar diblock copolymer system. (**a**) Limiting cases of lamellar alignment and orientation with respect to electrode surfaces lead to normalized effective conductivities that are maximum ($\sigma_{eff}=1$), random distribution averaged (2/3), and zero conductivity. (**b**) Degenerate alignments yield maximum ($\sigma_{eff}=1$) and weighted average ($\sigma_{eff}=1/2$) conductivities.

Further analysis of the model for a system composed of periodically alternating conducting and insulating slabs, or lamellae, leads to the conclusion that the effective conductivity of the system, $\sigma_{eff}$, is maximized for perpendicular orientation of lamellae with respect to current-supplying electrodes (i.e., for the situation in which the lamellar surface normal is perpendicular to the direction of current flow/lies in the plane of the electrodes). Conversely, for the orthogonal situation, i.e., for parallel arrangements of such lamellae, the effective conductivity is zero (Figure 1). In general we can write that $\sigma_{eff}$ depends on volume fraction of the conductive A block, $\varphi_{A}$, its intrinsic conductivity $\sigma_{A}$ (implicitly assumed to be morphology-independent) and the “alignment” factor *d*, with $\sigma_{eff}=d(\varphi_{A})\sigma_{A}$. Thus, for the all-perpendicular lamellar arrangement *d* = 1, while for the parallel orientation *d* = 0.

The above analysis can be extended to a system without macroscopic orientational order, i.e., one composed of a collection of randomly oriented lamellar domains. Assuming perfect connectivity between adjacent domains, or more formally, that grain boundaries do not impose significant resistance to ion transport, one can make an argument that in such a sample only 2/3 of lamellae contribute to ion conduction. The remaining lamellae, oriented parallel to electrodes, effectively acts as “dead ends” and do not facilitate charge transport. On the basis of this assumption, there is a potential for only a 50% increase in conductivity between a disordered and a perfectly aligned sample predicted by this model [2,4]. Experimental work by Chintapalli et al. suggests that the assumption of low grain boundary resistance is justified for lithium ion conduction in a poly(styrene-block-etylene oxide) block copolymer [19], and computational work by Shen et al. has recovered the 2/3 factor under such conditions [20]. Realizing the potential 50% increase requires global alignment of the ion conducting microdomains in the system. The assumption of perfect connectivity in the aforementioned model represents a best-case scenario. In the absence of perfect connectivity, one anticipates a morphology factor *d* < 2/3 for randomly oriented lamellae, and therefore, a greater than 50% improvement in conductivity for uniform perpendicularly aligned lamellae. We neglect here for the time being the question of microdomain persistence that manifests itself in finite grain size in polydomain samples, and as pathway discontinuities in the aligned cases. To maximize transport, the challenge is therefore to impart uniform perpendicular alignment of lamellar microdomains in BCP PEMs [21].

### 1.2. Magnetic Alignment of Ion-Conducting Block Copolymers

Lamellar-forming BCPs can be aligned by the application of uni-directional external fields (e.g., magnetic or electric fields) [22,23]. The director for the system, the axis about which the system has the highest rotational symmetry, is the lamellar surface normal, **n**. The limiting cases correspond to alignment of **n** either perpendicular or parallel to the applied field. The latter case produces a singular orientation of lamellae as the director is uniquely constrained. The former and all other cases represent degenerate scenarios in which a continuous distribution of lamellar orientations about a given field direction represent isoenergetic states. For the limiting case of **n** perpendicular to the field, the director is unconstrained as it can lie at any angle in the plane perpendicular to the applied field [24]. Such orientational degeneracy however does not pose a problem in optimizing the conductivity of a BCP PEM. As depicted in Figure 1b, the effective conductivity across the membrane is still maximized as long as all lamellae are oriented perpendicular to the electrodes. For brevity this arrangement will be referred to as “perpendicular”, whereas the orthogonal alignment will be called “parallel”. Using the previous argument, one anticipates that the effective conductivity of the degenerate parallel arrangement should be 0.5.

**Figure 2:**
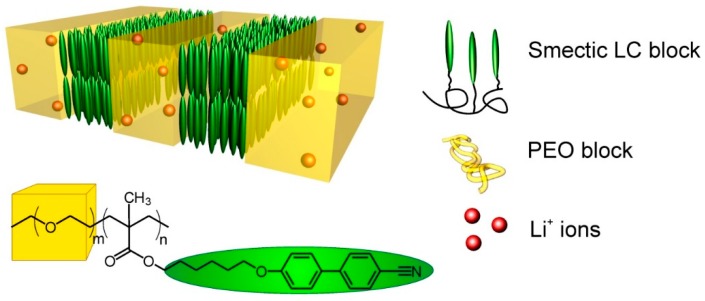
Schematic representation of lithium-doped block copolymer morphology and molecular structure of poly(ethylene oxide-b-6-(4-cyanobiphenyl-4-yloxy)hexylmethacrylate) PEO-*b*-PMA/CB.

Here, we examine the effect of lamellar alignment on the ionic conductivity of a BCP PEM, based on poly(ethylene oxide-b-6-(4’-cyanobiphenyl-4-yloxy)hexylmethacrylate) (PEO-*b*-PMA/CB, Figure 2). The polar PEO block acts as a host for lithium ions while the liquid crystalline (LC) PMA/CB block provides an effective lever for magnetic alignment of the lamellae. Prior studies of cylinder-forming PEO-PMA/CB revealed a 10-fold improvement in conductivity in field aligned samples, relative to those with randomly oriented cylindrical microdomains [25], whereas the microstructural model anticipates only a 3-fold improvement. Here, we consider degenerate perpendicular, degenerate parallel, and non-aligned lamellae at various Li concentrations. In contrast to the aforementioned findings, the improvement in conductivity due to alignment of lamellar microdomains is much more modest, in the order of 50% (a factor of 1.5X) in most cases. Perpendicular-aligned lamellae consistently display the highest conductivity, but the improvement relative to random and degenerate-parallel aligned lamellae decreases with increasing Li-concentration. Scattering experiments show that this decrease is principally due to a loss of alignment efficacy at higher Li concentrations.

## 2. Materials and Methods

### 2.1. Materials

Poly(ethylene oxide-b-6-(4′-cyanobiphenyl-4-yloxy)hexylmethacrylate), PEO(5.0 kg/mol)-b-PMA/CB (4.5 kg/mol) (PDI = 1.15) was supplied by Polymer Source (Montreal, QC, Canada). The material was purified from ionic impurities by triple extraction in a dichloromethane/water mixture, and then thoroughly vacuum dried at 100 °C. Final drying and all subsequent preparation steps including the assembly of conductivity, SAXS and DSC cells were performed inside a glovebox (<4 ppm O2, <1 ppm H_2_O). Lithium perchlorate (LiClO_4_) (battery grade, 99.99%) and dimethyl formamide (DMF) (anhydrous, 99.8%) were used, as received from Sigma-Aldrich (St. Louis, MO, USA).

### 2.2. Preparation of LiClO_4_ Doped Polymer Samples

Controlled amounts of a 0.7 wt % solution of LiClO_4_ were added drop wise into 5% aliquots containing 100 mg of polymer in DMF and cast onto glass Petri dishes. After initial solvent evaporation under ambient conditions, residual DMF was removed by placing samples under vacuum for 24 h at 60 °C and then at 100 °C for another 48 h.

### 2.3. Assembly of Conductivity and SAXS/WAXS Cells

Each cell consisted of two mirror polished aluminum plates separated by the PTFE spacer with the circular opening for the polymer sample forming a cell with a constant of 0.120 cm^−1^. The evenness of the filling of the cells was carefully observed by weighing the polymer and molding it in at the elevated temperature where it fills the space more evenly. The second electrode was gently pressed on top of the assembly to seal the cell. In case of SAXS cells the aluminum plates were substituted with two sheets of Kapton foil.

### 2.4. Magnetic Alignment

The magnetic alignment experiments were performed with the use of superconducting electromagnet at 5 T static magnetic field (American Magnetics Inc., Oak Ridge, TN, USA). The SAXS and conductivity cells were mounted on a theromostated aluminum block allowing the control of orientation of the sample with respect to the field. Two primary orientations were used: The first one where magnetic field direction is perpendicular to the surface of the electrodes and the second one where the field was applied in the parallel direction. These two are referred to as “perpendicular” and “parallel” alignments for brevity. In all runs the samples were subject to the same temperature profile starting with a brief ramp to 120 °C followed by slow cooling to room temperature at 0.1 °C/min rate. The “randomly” aligned samples were obtained by annealing them using the same procedure in the absence of the magnetic field. 

### 2.5. Small Angle X-ray Scattering

Small angle X-ray scattering (SAXS) experiments were carried out using a Rigaku S-MAX3000 system (The Woodlands, TX, USA) using pinhole collimation of Cu Kα radiation at 1.54 Å with a 2-D electronic wire detector giving access to a range of scattering vectors from 0.02 to 0.4 Å-1. Azimuthal integration of the scattered intensity to produce a 1D variation of intensity as a function of scattering vector q was performed using MATLAB routines (Rigaku,). q = (4π/λ) sinθ, with 2θ the scattering angle. SAXS data were calibrated using silver behenate standard with d-spacing of 58.38 Å. Temperature dependent measurements were done using a hot stage (Linkam THMS600, Tadworth, UK) with an associated temperature controller. For temperature dependent scattering, the sample chamber was evacuated and then refilled with helium to provide a low scattering atmosphere that aided heat transfer between the hot stage and the polymer films. Samples were subjected to a heating rate of 2 °C/min and allowed to equilibrate for 10 minutes at each temperature prior to data acquisition.

### 2.6. Differential Scanning Calorimetry

DSC pans were loaded and sealed inside the glovebox. The thermal behavior of the system was studied using DSC Q200 (TA instruments, New Castle, DE, USA) at a heating rate of 20 °C/min between −75 and 110 °C.

### 2.7. Conductivity Measurements

After magnetic alignment the sample cells were mounted on a theromostated two electrode stage connected to Solartron 1260 analyzer (Solartron Analytical, Farnborough, UK) operating in 0.1–10^6^ Hz frequency range at the amplitude of 100 mV. Sample conductivity was calculated using the value of electrical resistance obtained by fitting the data to the equivalent circuit model with the ZPlot^®^ software (Solartron). The isothermal measurements were performed outside of the magnet after the thermal runs were accomplished. Conductivity temperature scans were performed in 3 °C increments at 0.5 °C/min heating rate with an equilibration time of 3 min at each measurement point.

### 2.8. Atomic Force Microscopy (AFM)

AFM was performed using a Multimode AFM (Bruker, Santa Barbara, CA, USA) in tapping mode (phase imaging) in air with standard Si probes of f = 350 kHz (TESPA, Veeco). Samples were prepared by exposing the cross-sections using a Leica EM ultramicrotome equipped with a diamond knife.

## 3. Results and Discussion

### 3.1. Thermal and Morphological Characterization

**Figure 3:**
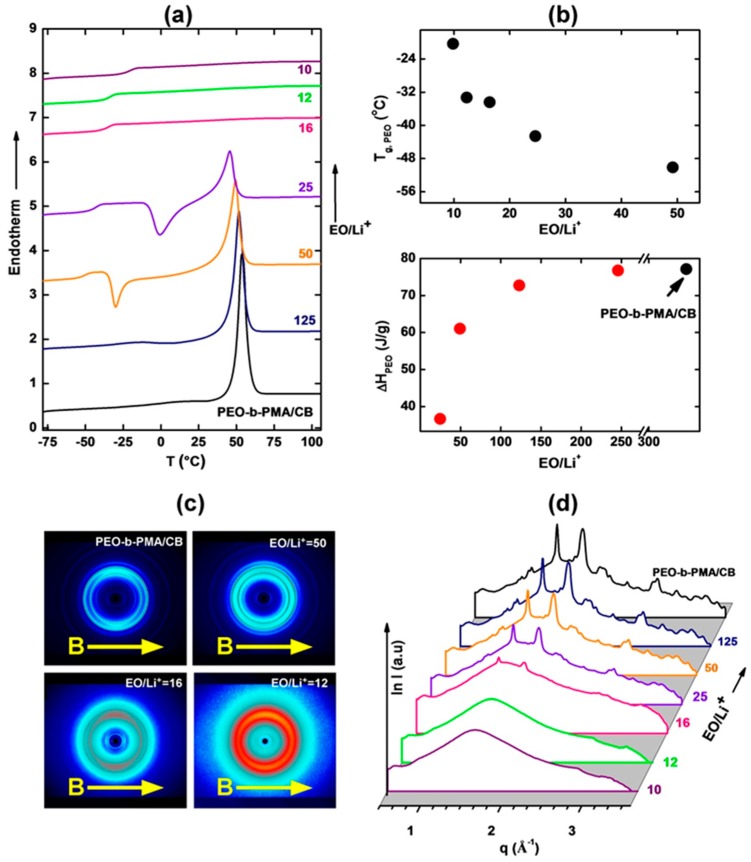
(**a**) DSC data of the PEO-b-PMA/CB system at different Li-doping levels as indicated (**b**) Corresponding glass transition temperature of PEO and PEO melting enthalpy of the system. Data clearly indicate a glass transition temperature increase and suppression of PEO crystallization with the inclusion of Li^+^. The DSC data are corroborated by the 2D WAXS data shown in (**c**). (**d**) Circularly integrated WAXS data for different EO:Li^+^ ratios. PEO diffraction peaks display uniform azimuthal distribution indicating lack of preferential orientation of the PEO chains in the field-aligned samples.

The lamellar system studied here has a total molar mass of 9.5 kg/mol with a PEO block mass fraction of 0.53. The DSC data presented in Figure 3a indicate that PEO is present in a semi-crystalline form, and that the degree of crystallinity decreases with increasing concentration of the Li salt. While the crystalline PEO fraction in the neat BCP (i.e., without Li) is estimated to be about 36% based on the magnitude of PEO melting enthalpy relative to that of pure highly crystalline PEO [26,27], the glass transition temperature for the neat material was not observed. Glass transitions were observed however in the Li-containing samples, with an increase in *T*_g_ recorded with increasing Li-concentration (Figure 3b). These observations indicate a decrease in the crystallinity of PEO on Li inclusion, and are consistent with the expected complexation of lithium ions by ether oxygen atoms of the PEO backbone [12,28]. The LC phase transitions in the PMA/CB block could not be resolved by DSC for all samples studied, a small endothermic LC clearing transition peak was observed for the neat diblock. The DSC data are corroborated by WAXS measurements (Figure 3c,d) where crystalline PEO diffraction peaks can be seen up to 16:1 EO:Li^+^ ratio. For this particular stoichiometry, we observed a delay in PEO crystallization ranging from several days up to two weeks after complete PEO melting above the ODT temperature and subsequent cooling to room temperature.

**Figure 4:**
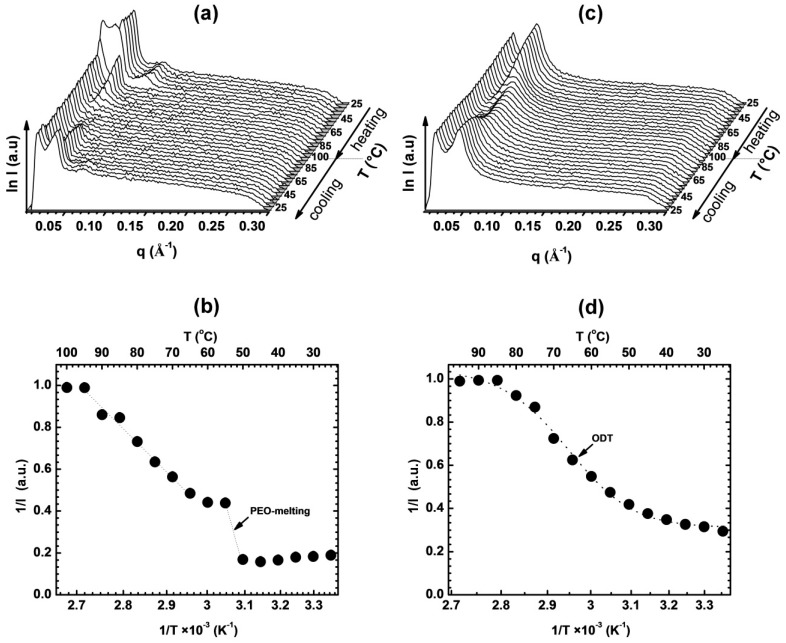
Temperature dependent SAXS data and the corresponding inverse intensities as a function of inverse temperature for the neat diblock PEO-b-MA/CB (**a**, **b**) and for a sample with EO:Li^+^ = 16 (**c**, **d**). For the neat diblock, ODT is followed by PEO crystallization. For EO:Li^+^ = 16, the system is amorphous and no changes in scattering intensity are observed on cooling below ODT. Dotted lines are visual guides.

Temperature resolved SAXS permitted characterization of the phase behavior of the block copolymer and therefore optimization of the temperature range used in subsequent magnetic alignment experiments. The non-aligned neat BCP scattering pattern displays two peaks at characteristic q* and 2q* locations, attributable to lamellar morphology (Figure 4a). At the melting point of PEO (approx. 55 °C), the intensity of both peaks abruptly decreases (Figure 4b) and ultimately disappears at about 85 °C where the sample undergoes the order-disorder transition. In case of Li-containing samples, in particular at higher Li concentrations, we observed a broadening of the ODT temperature range (Figure 4c). While a smectic A mesophase with a d-spacing of ~3.5 nm is known to form by LC assembly of the cyanobiphenyl mesogens of the PMA/CB block, the associated SAXS reflections were too weak to be clearly resolved in the scattering data of non-aligned samples.

**Figure 5:**
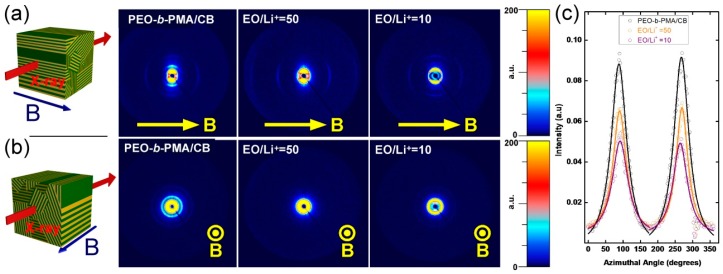
(**a**) Room temperature 2D SAXS data of EO-MA/CB samples at different EO:Li^+^ ratios, where the field was kept horizontal with respect to the X-ray detector’s plane (top panel) and the corresponding azimuthal intensity profiles of the BCP super structure peak (**c**). (**b**) Room temperature 2D SAXS data of the same samples when the field was applied normal with respect to the X-ray detector’s plane (bottom panel).

SAXS data from samples aligned in the magnetic field delivered further information about the self-assembled structure of the system. In the alignment experiments we used the same thermal annealing procedure for all samples, consisting of clearing the microstructure at 120 °C (thereby providing a broad margin above the T_ODT_ of all samples) followed by slow cooling (0.1 °C/min.) in the presence of a static 5 tesla (T) magnetic field. 2D SAXS data collected with X-rays incident perpendicular to the direction of the applied field reveal a high degree of alignment with lamellar microdomains oriented along the field axis (lamellar normal perpendicular to the field) indicated by scattering along the vertical direction, with scattering peaks up to fourth order resolvable (Figure 5a). Correspondingly, the smectic A layers within the PMA/CB block are aligned perpendicular to the field (LC director is parallel to the field), as inferred from the intense reflections along the equatorial direction of the scattering data.

**Figure 6:**
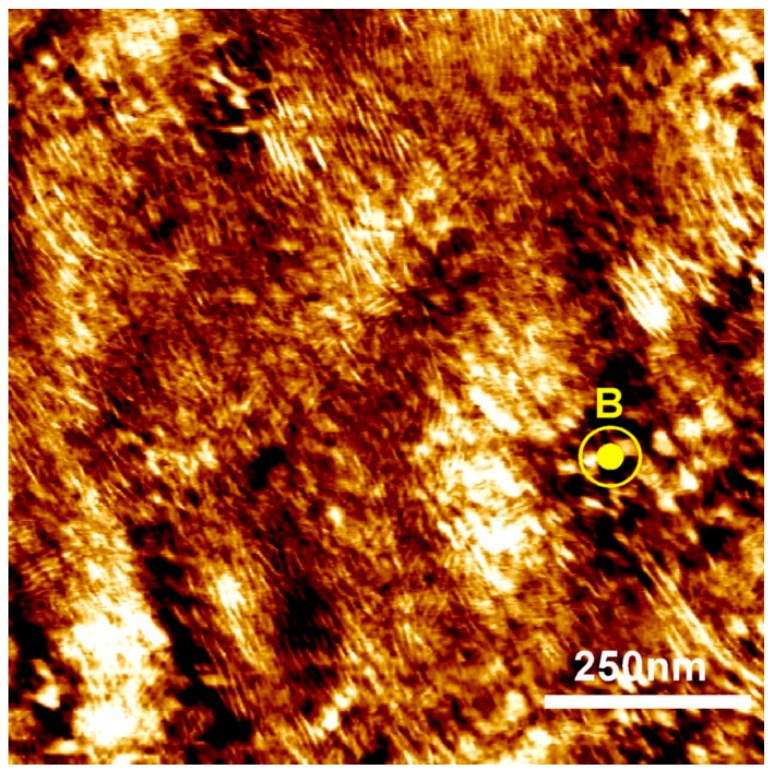
AFM image of PEO-b-PMA/CB cross section aligned in 6 T magnetic field applied in the direction perpendicular to the image plane.

The data are consistent with the positive diamagnetic anisotropy of the cyanobiphenyl moiety, and planar anchoring of these mesogens at the inter-material dividing surface (IMDS) between the PEO and PMA/CB blocks. These conditions result in an orthogonal relationship between the orientation of diblock lamellar microstructure and smectic liquid crystalline layers (Figure 1). Such geometric constraints, however do not lead to a unique spatial orientation of the lamellae, but to a degenerate alignment where the orientation of domains of equivalent energy is symmetric about the axis of the applied field. The degeneracy of the alignment is clearly seen on SAXS panels presented in Figure 5b where the X-rays are incident parallel to the field direction. The lamellar microstructure scattering can be seen as a set of concentric rings with intensity uniformly distributed along the azimuthal angle of the detector. Conversely, the smectic reflections are out of the Bragg condition and therefore absent in such a scattering geometry. The degeneracy of the alignment is further supported by the atomic force microscopy image (Figure 6) performed on the cross-section of a sample aligned at 6 T, with the sample cut in a direction perpendicular to the magnetic field, i.e., to provide a cross-sectional view along the field direction, as indicated. The sample is polydomain with lamellae displaying “edge-on” orientation.

The quality of the alignment of the samples with various lithium content can be deduced from the analysis of the width of azimuthally integrated SAXS data (Figure 5c). The full-width at half-height (fwhm) of the (001) lamellar peak increases with lithium concentration level. The decrease in the quality of alignment with Li concentration is likely due to a reduction of PEO chain mobility that slows the field response of the system during the fixed cooling rate ramp across the ordering transition.

**Figure 7:**
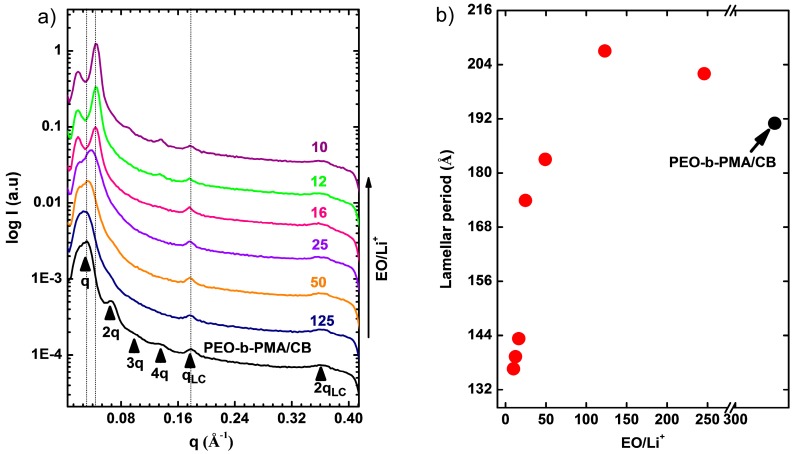
(**a**) Circularly integrated data of PEO-b-PMA/CB samples at different EO:Li^+^ ratios. The samples preserve lamellar morphology at all LiClO_4_ doping levels. (**b**) The lamellar period decreases with doping level above 150:1 EO:Li^+^ ratio, while the smectic layer spacing remains unchanged.

Circularly integrated SAXS data of aligned samples with different LiClO_4_ content are presented in Figure 7a. The presence of the Li salt does not trigger morphological transformations and all samples remain lamellar. The lamellar repeat distance, however, is a sensitive function of lithium concentration. The lamellar d-spacing initially increases with lithium content from about 19 nm, reaching a maximum of roughly 21 nm at a stoichiometry of 150:1 EO:Li^+^. The increase in d-spacing is consistent with chain stretching due to increase in the block segregation strength on Li addition, as seen previously in other systems [3,29,30,31]. However, for higher lithium loadings (i.e., for stoichiometric ratios below 150:1 EO:Li^+^), the d-spacing decreases dramatically, reaching a minimum value of about 13.5 nm at the highest Li content at a stoichiometry of 10:1 EO:Li^+^. The decreasing d-spacing appears to be related to the loss of crystallinity of PEO at Li concentrations above 150:1 EO:Li^+^, as inferred from DSC data (Figure 3). 

Such a decrease of d-spacing due to loss of crystallinity has been observed in semicrystalline block copolymers [32,33], but very strong couplings between crystallinity and d-spacing (large changes of d-spacing) are typically linked to so-called breakout crystallization in which the crystallization significantly disrupts the block copolymer superstructure [34]. We note in passing that there is no evidence that the current system exhibits such breakout crystallinity, as inferred by the preservation of characteristic lamellar scattering across all temperatures. It is apparent that the effects of crystallinity compete with those due to an increase in segregation strength on Li addition-increasing segregation strength on Li-addition favors a larger d-spacing while suppression of PEO crystallinity by Li favors a lower d-spacing. By contrast, the d-spacing of the smectic A layers of the PMA/CB block (3.5 nm) is invariant with Li concentration. This d-spacing invariance of the smectic layers suggests that Li is not present in the LC, but instead partitions very selectively into the PEO block.

### 3.2. Ionic Conductivity

**Figure 8:**
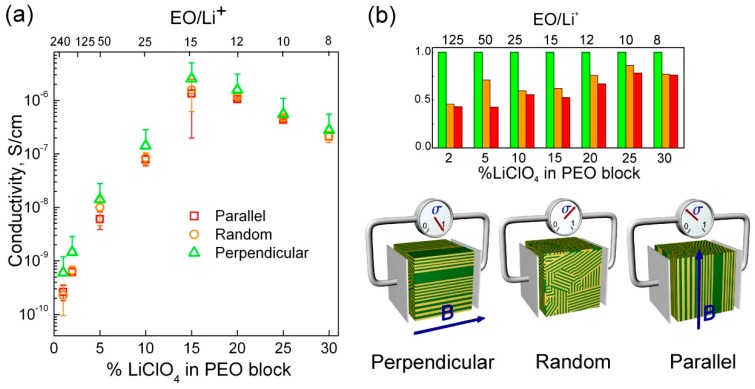
(**a**) Room temperature conductivities as a function of lamellar domains orientation shown for samples with different ethylene oxide units to Li ion ratio. (**b**) Linear representation of conductivity data normalized to the conductivity of samples with perpendicular domains orientation (green bars). The error bars represent standard deviation of at least three independently repeated measurements for each data point.

The room temperature conductivities of samples in different orientations as a function of Li concentration are shown in Figure 8a on a semi-logarithmic plot. The different orientations of magnetically aligned lamellae are for simplicity named after the direction of the field with respect to the plane of the electrodes. In this terminology, “perpendicular” refers to the degenerate perpendicular situation referenced earlier. For all lamellar orientations, the conductivity increases steeply with increasing Li concentration up to a stoichiometric ratio of 15:1 EO:Li^+^ beyond which it falls off slowly. The decrease in conductivity despite increasing Li concentration is likely due to ion-pairing as commonly encountered at elevated Li concentrations in solid polymer electrolytes [35,36,37,38]. The relative conductivities of samples aligned as a function of orientation (perpendicular, parallel, and “random” or non-aligned) can be better visualized on the linear plot in Figure 8b. In the perpendicular arrangement all PEO domains are effectively in the conductivity-maximizing orientation so the value of the conductivity of these samples was used to normalize the conductivities of the random and parallel-aligned samples.

The values measured for 125:1 EO:Li^+^ sample are accompanied by a relatively large experimental uncertainty due to the difficulty of accurately determining the very high electrical impedance of these samples. Concurrently, for 15:1 EO:Li^+^, the large variability in the time associated with PEO crystallization led to uncertainty in conductivity values due to difficulty in consistently reproducing the same state of the sample during the conductivity measurements. Nevertheless, several clear trends emerge. The improvement in conductivity realized by perpendicular alignment (relative to random) was roughly 2-fold for the 4 lowest Li-concentration samples, i.e., those of stoichiometric ratios from 125:1 to 15:1 EO:Li^+^. Beyond 15:1 EO:Li^+^, the ratio of perpendicular to random lamellar conductivity decreased, falling to roughly 1.33X at the highest Li-concentration (8:1 EO:Li^+^). The ratios of conductivity measured for the 50:1 EO:Li^+^ sample were roughly σ_‖_/σ_┴_ = 0.50 and σ_rand_/σ_┴_ = 0.66. These values are identical to those predicted by the microstructural model, but these ratios increased to roughly 0.75 at higher lithium concentrations. We speculate that the increases originate simply due to the decreasing quality of alignment observed in samples on increasing Li concentration. The precise reason for the loss of alignment quality is unclear. One possibility is simply that the kinetics of the alignment are poorer at larger Li loadings.

**Figure 9:**
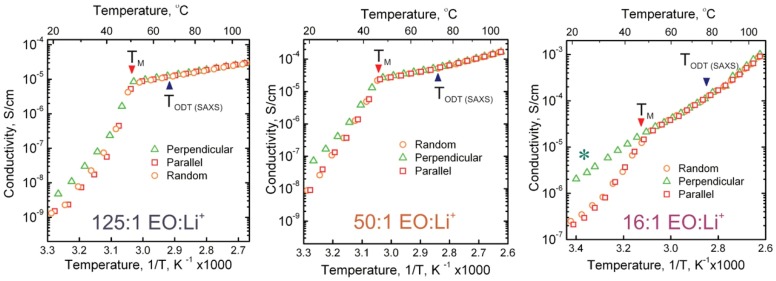
Temperature dependence of conductivities for perpendicular, parallel and random lamellae at 125:1, 50:1 and 16:1 EO:Li^+^ stoichiometries, as indicated. *T*_M_ and T_ODT_ mark the locations of PEO melting and the order-disorder transition inferred from DSC and SAXS data respectively. *Note in the case of the 16:1 perpendicular lamellae that the PEO remained amorphous during the measurement.

Figure 9 shows the evolution of ionic conductivity for three selected samples with temperature, in the Arrhenius convention. Surprisingly, contrary to prior findings in a cylinder-forming system, there is no clear signature of the order-disorder transition (marked with a blue triangle)—the conductivity evolves smoothly, monotonically increasing with temperature through the ODT. Instead, the conductivity data are dominated by the PEO melting transition and all three plots show a clear break in the scaling of conductivity near the melting temperature, *T*_M_, of PEO, from which we can infer a significant decrease in the activation energy for ionic transport in the PEO melt compared to the PEO crystal. Similar strong dependence of activation energy on electrolyte backbone crystallinity has been reported [39]. In the case of 50:1 EO:Li^+^ there is approximately a 7-fold reduction in the activation energy from ~39 k_B_T_298_ (~1 eV) to 5.5 k_B_T_298_ (~0.14 eV). The activation energy in the amorphous state was similar for 125:1 EO:Li^+^ at ~6 k_B_T_298_, but was significantly higher in the amorphous state for 16:1 EO:Li^+^, at ~12 k_B_T_298_, highlighting the significant reduction in conductivity due to likely ion-pairing at this Li concentration. Moreover, the conductivities of the samples with perpendicular, parallel and random domain orientation follow similar monotonic curves towards the melting point of PEO, at which point any difference between the absolute values of their conductivity becomes minimal. The conductivities for the three alignments effectively merge at this temperature, even though the alignment and the nanostructure of the block copolymer are preserved well beyond PEO *T*_M_, i.e., up to the BCP ODT temperature. The order-disorder transition, unlike in the case of cylindrical system, cannot be recognized from conductivity plots. This finding is, however, consistent with the behavior of other PEO-containing block copolymer membranes reported by other researchers.

The values of conductivity ratios measured at room temperature are broadly consistent with the predictions of the microstructural model described here, and are in line with those observed in prior work by Park and Balsara [2]. The temperature dependence of conductivity was rather surprising, given the previously observed behavior of the chemically analogous cylinder-forming material. The disparity appears to be due to the differing nature of PEO crystallization in these systems. The data clearly indicates that such crystallization plays a critical role in the lamellar system, while in the cylindrical system PEO was amorphous for all compositions in the same temperature range. The difference in the crystallization behavior can be explained by both the larger molar mass of PEO in the lamellar sample and the weaker geometrical constrains for the folding of PEO chains during crystallization inside lamellar microdomains compared to chain folding in cylindrical confinement. 

The experimental evidence (SAXS) indicates that the samples remain aligned at temperatures above PEO melting and that PEO crystallization is not necessary for field alignment (samples with amorphous PEO at higher LiClO_4_ content were also aligned). Nevertheless, we investigated whether the differences in conductivity could be attributed to the effect of the magnetic field on PEO crystallization. Prior work has reported moderate texturing of crystalline PEO domains for PEO-containing block copolymer samples that underwent crystallization in the presence of large magnetic fields [40]. As a control experiment, samples were field-cooled to 60 °C at the usual rate. The field was then switched off for the second portion of the cooling ramp to room temperature, allowing PEO crystallization to take place in the absence of the field. The conductivities of samples prepared in this manner were the same as those for samples that underwent PEO crystallization in the presence of the field. This indicates that the observed conductivity characteristics originate from the macroscopic texturing of the sample by microdomain orientation, and are not the effect of a field-driven alteration of the PEO conductivity by PEO chain alignment, or any other field-related effect. This assertion is consistent with the 2D WAXS data (Figure 3c) in which the PEO diffraction rings display azimuthally uniform intensity, indicating lack of preferential orientation of the PEO chains in the field-aligned samples.

The possibility exists that the anisotropy of the conductivity originates here solely due to preferential anisotropic orientation of PEO crystallites confined by the magnetically aligned BCP superstructure. This effect would tend to diminish on decreasing the degree of crystallinity of the conductive block, as observed here for samples at higher Li concentration. However, the fact that the alignment quality also decreases with Li concentration for kinetic reasons makes it difficult to decouple these effects. We speculate that the dominant factor is the reduction of microdomain alignment efficacy at higher Li concentrations.

## 4. Conclusions

In summary, we have shown how the macroscopic orientation of lamellar microdomains influences bulk ionic conductivity of a liquid crystalline block copolymer membrane. We presented a method to generate anisotropically-conductive materials with strongly, yet degenerately, aligned microstructure. The room temperature ionic conductivity along the field direction is about two times higher than that in the perpendicular direction, and about 50% greater than the conductivity of randomly oriented or non-aligned samples. These numbers are in rough agreement with the model developed for transport of small molecules in microstructured lamellar block copolymers. The validity of the model depends on several conditions, including full connectivity between adjacent grains. Relatively free flow of charge across macroscopic distances between electrodes is likely facilitated by the orientational degeneracy of alignment that produces a coexistence of many differently oriented microdomains. Charge transport is also likely facilitated by the presence of numerous defects connecting the grains; both of these features are visible in the AFM micrograph of a representative sample.

Our current findings differ from prior observations of a cylinder-forming system of similar chemical composition. In that case, the relative conductivities were much higher and deviated strongly from expectations of the microstructural model due to the lack of the connectivity between the conductive minority phase microdomains. Moreover, the pronounced differences in conductivity above and below PEO *T*_M_ for the lamellar system relative to the cylinder-forming one indicates the important role of PEO crystallization in the anisotropic conductivity of the lamellar system. More broadly, the ability to create global alignment of lamellar or cylindrical microdomains in ion conducting block copolymer samples is significant in the context of understanding the transport mechanisms in these heterogeneous systems, verifying and examining the validity of theoretical predictions, and processing BCP membranes with optimized ionic conductivities.
